# Clinical Outcome of CT-Guided Stereotactic Ablative Brachytherapy for Unresectable Early Non-Small Cell Lung Cancer: A Retrospective, Multicenter Study

**DOI:** 10.3389/fonc.2021.706242

**Published:** 2021-09-15

**Authors:** Zhe Ji, Bin Huo, Shifeng Liu, Qinghua Liang, Chao Xing, Miaomiao Hu, Yanli Ma, Zhe Wang, Xinxin Zhao, Yuqing Song, Yufeng Wang, Hongmei Han, Kaixian Zhang, Ruoyu Wang, Shude Chai, Xuequan Huang, Xiaokun Hu, Junjie Wang

**Affiliations:** ^1^Department of Radiation Oncology, Peking University Third Hospital, Beijing, China; ^2^Department of Thoracic Surgery/Department of Oncology, The Second Hospital of Tianjin Medical University, Tianjin, China; ^3^Department of Intervention Therapy, The Affiliated Hospital of Qingdao University, Qingdao, China; ^4^Center of Minimally Invasive Intervention, Southwest Hospital of Army Medical University (The First Hospital Affiliated to the Army Medical University), Chongqing, China; ^5^Department of Oncology, Tengzhou Central People’s Hospital, Tengzhou, China; ^6^Department of Oncology, Staff Hospital of Chengde Iron and Steel Group Co. Ltd., Chengde, China; ^7^Department of Radiation Oncology, Affiliated Zhongshan Hospital of Dalian University, Dalian, China; ^8^Department of Oncology Radiotherapy, The First People’s Hospital of Kerqin District, Tongliao, China; ^9^Department of Nuclear Medicine, Xuzhou Cancer Hospital, Xuzhou, China

**Keywords:** early non-small cell lung cancer, stereotactic ablative brachytherapy, radioactive seed implantation, efficacy, prognostic factors

## Abstract

**Objective:**

To analyze the efficacy and safety of low dose rate stereotactic ablative brachytherapy (L-SABT) for treatment of unresectable early-stage non-small cell lung cancer (NSCLC).

**Methods:**

Data of patients with early-stage NSCLC who received CT-guided L-SABT (radioactive I-125 seeds implantation) at eight different centers from December 2010 to August 2020 were retrospectively analyzed. Treatment efficacy and complications were evaluated.

**Results:**

A total of 99 patients were included in this study. Median follow-up duration was 46.3 months (6.1-119.3 months). The 1-year, 3-year, and 5-year local control rates were 89.1%, 77.5%, and 75.7%, respectively. The 1-year, 3-year, and 5-year overall survival rates were 96.7%, 70.1%, and 54.4%, respectively. Treatment failure occurred in 38.4% of patients. Local/regional recurrence, distant metastasis, and recurrence combined with metastasis accounted for 15.1%, 12.1%, and 11.1%, respectively. Pneumothorax occurred in 47 patients (47.5%) with 19 cases (19.2%) needing closed drainage. The only radiation-related adverse reaction was two cases of grade 2 radiation pneumonia. KPS 80–100, T1, the lesion was located in the left lobe, GTV D90 ≥150 Gy and the distance between the lesion and chest wall was < 1 cm, were associated with better local control (all *P* < 0.05); on multivariate analysis KPS, GTV D90, and the distance between the lesion and chest wall were independent prognostic factors for local control (all *P* < 0.05). KPS 80–100, T1, GTV D90 ≥150 Gy, and the distance between the lesion and chest wall was < 1 cm were also associated with better survival (all *P* < 0.05); on multivariate analysis KPS, T stage, and GTV D90 were independent prognostic factors for survival (all *P* < 0.05). The incidence of pneumothorax in patients with lesions <1 cm and ≥1cm from the chest wall was 33.3% and 56.7%, respectively, and the differences were statistically significant (*P* = 0.026).

**Conclusion:**

L-SABT showed acceptable efficacy in the treatment of unresectable early-stage NSCLC. But the incidence of pneumothorax is high. For patients with T1 stage and lesions <1 cm from the chest wall, it may have better efficacy. Prescription dose greater than 150 Gy may bring better results.

## Introduction

Surgery is the main treatment for early non-small cell lung cancer (NSCLC). For unresectable early NSCLC, stereotactic ablative radiotherapy (SABR) is considered the best option (1). However, in the real-world clinical practice, the situation can be more complex. There are also some patients who cannot be operated on who also did not receive external beam radiotherapy (EBRT) because of various reasons. For these patients, the prognosis is poor, with a 5-year survival rate of less than 10% (2). With the development of clinical practice, radioactive I-125 seed implantation (RISI) is more widely used in the local treatment of tumors. This method is to implant the radioactive I-125 seeds into the tumor, and the tumor cells will be killed by continuous gamma ray irradiation generated by the seeds (3). In view of the high dose and low fraction of brachytherapy, we also called RISI as low dose rate stereotactic ablative brachytherapy (L-SABT) (4). L-SABT has been reported to be a safe and effective treatment for various solid tumors (5–8), which provides another treatment option for the clinic. However, L-SABT has rarely been used for the treatment of early-stage tumors (except prostate cancer), and so there is limited data about its efficacy in these cases. This study retrospectively analyzed the data of patients with early NSCLC who received L-SABT, in order to further clarify the clinical efficacy and safety of L-SABT and provide data for the actual clinical practice of the real world.

## Material and Methods

### Patients

Due to the small number of patients receiving L-SABT for early stage NSCLC, this study combined the data of 8 medical centers between December 2010 and August 2020. Case selection criteria include: (1) they had received pathologically confirmed diagnosis of NSCLC (squamous cell carcinoma or adenocarcinoma, excluding other types of NSCLC) at first visit; (2) they had stage T1–3N0M0 (stage Ia–IIb) based on the UICC TNM classification 8th edition (9) after systemic evaluation (evaluation methods include CT/MRI and/or PET-CT), the re-staging was carried out for cases before 2016; (3) they were not suitable for surgery after being evaluated by an experienced thoracic surgeon or pulmonologist; (4) L-SABT had been used as the initial treatment; (5) D90 (dose that covers 90% of target volume) had been ≥100 Gy on post-treatment evaluation. A total of 99 patients satisfied these criteria and were included in this study.

L-SABT treatment was conducted after obtaining informed consent from patients and their families. This retrospective study has been approved by the ethical committee.

### Devices and Instruments

(1) CT: Brilliance Bigbore CT, Philips. (2) I-125 seeds: type 6711_1985, from HTA Co., Ltd, with a half-life of 59.4 days and dose rate constant of 0.965 cGy/(h·U). (3) Radioactive I-125 seed implantation devices: Mick Radio-Nuclear Instruments and Eckert & Ziegler BEBIG. (4) Brachytherapy treatment planning system (BTPS): KLSIRPS-3D, Beijing University of Aeronautics and Astronautics and Beijing Astro Technology LTD, CO., which can calculate and display dose distribution of the target area and generate a dose-volume histogram (DVH). Planning system source data originated from the official, supplementary and reports, and update of the American Association of Physicists in Medicine (AAPM) (10–12).

### Preoperative Planning

Enhanced CT scan, with 5-mm slice thickness, was performed within 1 week before seed implantation. The imaging data were transmitted to the treatment planning system for preoperative evaluation and plan design. The treatment planning design involved delineation of gross tumor volume (GTV) and organs at risk (OARs); determination of prescription doses and seed radioactivity; determination of the puncture needle direction, distribution, and depth of insertion; determination of seed quantity; and simulation of the spatial distribution of seeds. The prescription dose was empirically set as ≥100 Gy.

### Seeds Implantation

Seeds implantation was performed under 1% lidocaine infiltration anesthesia. The disposable seed implantation needle was inserted into the target lesion under CT guidance. The needle tip was positioned 0.5 cm from the distal tumor margin. The space between each row of needles and between each seed was 0.5–1.0 cm. CT scan was performed after seeds implantation to make sure that the seeds distribution was as per the treatment plan. When necessary, additional seeds were implanted to avoid dosimetric cold spots.

### Postoperative Management and Dose Verification

**Figure 1:**
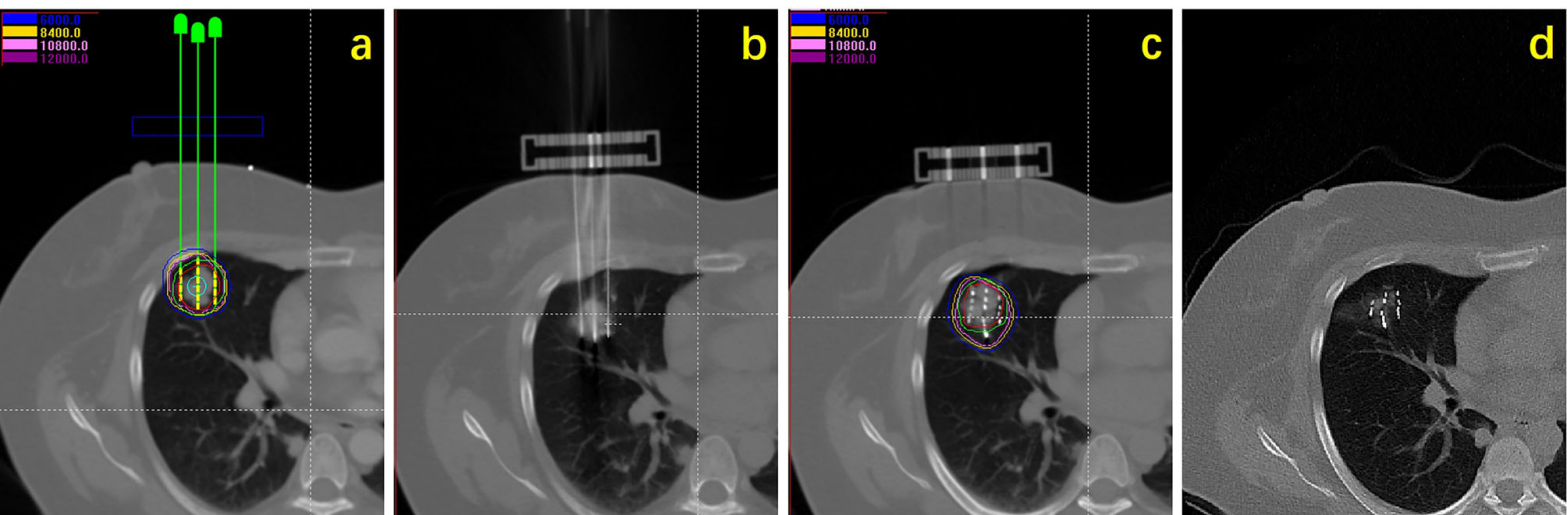
Flow chart showing procedure of seeds implantation: **(A)** Preoperative planning design; **(B)** intraoperative needle insertion; **(C)** seeds implantation and dose verification; **(D)** efficacy observation.

All patients received anti-infection and hemostasis treatment after implantation. Chest CT scan was performed 24 hours after operation to rule out pneumothorax, hemorrhage, and other complications. Postoperative dose verification was performed (**Figure 1**).

### Evaluation Indices

The main evaluation index was the local control rate. The secondary evaluation indices were overall survival, adverse reactions, and failure reasons. CT scan was used to detect tumor size changes during follow-up. The International Response Evaluation Criteria in Solid Tumor (RECIST) were used to evaluate treatment response (13). Complete response was defined as complete disappearance of the target tumor. Partial response was defined as a decrease of target lesion diameter to ≤30% of that at baseline. Progressive disease was defined as target lesion diameter increase by ≥20% or the appearance of new lesions. Stable disease was defined as any change intermediate between partial response and progressive disease. Puncture complications and radiation-related adverse reactions were graded according to Common Terminology Criteria for Adverse Events (CTCAE) version 5.0 (14); there were five grades, as follows: minor/grade 1 (no symptoms and no treatment required), moderate/grade 2 (symptoms present and treatment required), severe/grade 3 (symptoms not controlled by drugs, and instrumentation or invasive procedure required), life-threatening/grade 4 (emergency treatment required), and death/grade 5.

The factors assessed for influence on prognosis included the following: sex, KPS score, stage, pathological type, lesion location, and GTV D90 (dose received by 90% of GTV).

### Statistical Analysis

SPSS 25.0 (IBM Corp., Armonk, NY) was used for statistical analysis. Measurement data was expressed in median value (range) or mean value ± standard deviation and numeration data was expressed in absolute value and/or percentage value. The chi-square test was used to compare rates between groups. The Kaplan–Meier method was used to calculate the local control rate and survival rate. Log rank test was used for univariate analysis and Cox regression analysis was used for multivariate analysis. *P* ≤ 0.05 was considered statistically significant.

## Results

### Patients

A total of 99 patients were included in this study. The median age was 69.8 ± 9.06 years old (range, 49–91 years old). The median Karnofsky Performance Status (KPS) score was 80 (range, 60–100). Clinical stages were 47 cases (47.5%) of T1N0M0, 37 cases (37.3%) of T2N0M0, and 15 cases (15.2%) of T3N0M0. Pathological types were 45 cases (45.5%) of squamous cell carcinoma and 54 cases (54.5%) of adenocarcinoma. Because of the differences in patients’ economic conditions and medical conditions in local hospitals, 21 patients (21.2%) were staged by CT/MRI and the remaining 78 patients (78.8%) were staged by PET-CT.

### Seeds Implantation

Median lesion diameter was 3.2 ± 1.22 cm (range, 1.1-6.4 cm). The median number of seeds implanted was 41.8 ± 20.97 (range, 9-110). Median seeds radioactivity was 0.7 ± 0.07 mCi (range, 0.6–0.8 mCi). Median number of needles was 8 ± 4 (range, 2–25). Median postoperative GTV D90 was 165.8 ± 41.08 Gy (range, 110.4–278.8 Gy).

### Treatment Response

At the end of February 2021, the median follow-up duration was 46.3 months (range, 6.1-119.3 months). The 1-year, 3-year, and 5-year cumulative survival rates for the whole group were 96.7%, 70.1%, and 54.4%, respectively. The 1-year, 3-year, and 5-year progression-free survival rates were 79.5%, 61.10%, and 52.7%, respectively. The 1-year, 3-year, and 5-year local control rates were 89.1%, 77.5%, and 75.7%, respectively. Thirty-five patients (35.4%) died, and 64 patients (64.6%) survived. A total of 38 patients experienced treatment failure, including 10 cases (10.1%) of local recurrence, 4 cases (4.0%) of reginal recurrence, 12 cases (12.1%) of distant metastasis, 1 case (1.0%) of local recurrence with reginal recurrence, 9 cases (9.1%) of local recurrence with distant metastasis, and 2 cases (2.0%) of reginal recurrence with distant metastasis.

Procedure-related complications included pneumothorax, subcutaneous emphysema, hemothorax, hemoptysis, and seeds migration. Pneumothorax was the most common complication, with an incidence of 47.5% (47/99 patients). There were 19 (19.2%) patients with pulmonary compression volume >30%, and they were treated with invasive closed drainage, and all recovered. Most of the complications were grade 1, and only a few were grade 2. There were no grade 3-5 complications (**Table 1**).

**Table 1:** Complications.

Complications	N	%
Pneumothorax		
No	52	52.5
Yes	47	47.5
Subcutaneous emphysema		
No	96	97.0
Grade 1	3	3.0
Hemothorax		
No	86	86.9
Grade 1	13	13.1
Hemoptysis		
No	67	67.7
Grade 1	30	30.3
Grade 2	2	2.0
Radiation pneumonitis		
Grade 0-1	97	98.0
Grade 2	2	2.0
Seed shifting		
No	97	98.0
Yes	2	2.0

Radiation-related adverse reaction was seen in only 2 patients (2.0%) with grade 2 radiation pneumonia. No patient had skin reaction, esophagitis, myelitis, or other visible side effects (**Table 1**).

### Factors Affecting Outcomes

**Figure 2:**
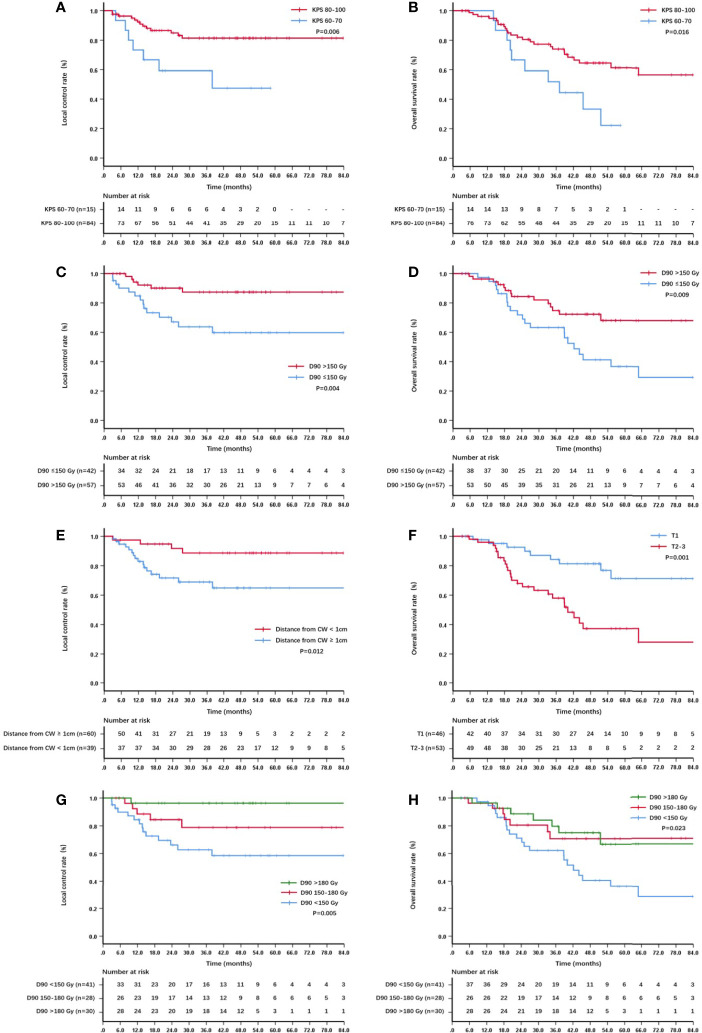
Kaplan–Meier curve about local control and survival: **(A)** The local control of patients with KPS score 60-70 and 80–100; **(B)** the overall survival of patients with KPS score 60-70 and 80–100; **(C)** the local control of patients with GTV D90 <150 Gy and ≥150 Gy; **(D)** the overall survival of patients with GTV D90 <150 Gy and ≥150 Gy; **(E)** the local control of patients with lesions <1 cm and ≥1cm from the chest wall; **(F)** the overall survival of patients with T1 and T2-3; **(G)** the local control of patients with D90 <150 Gy, 150-180 Gy,and >180 Gy; **(H)** the overall survival of patients with D90 <150 Gy, 150-180 Gy and >180 Gy.

**Figure 3:**
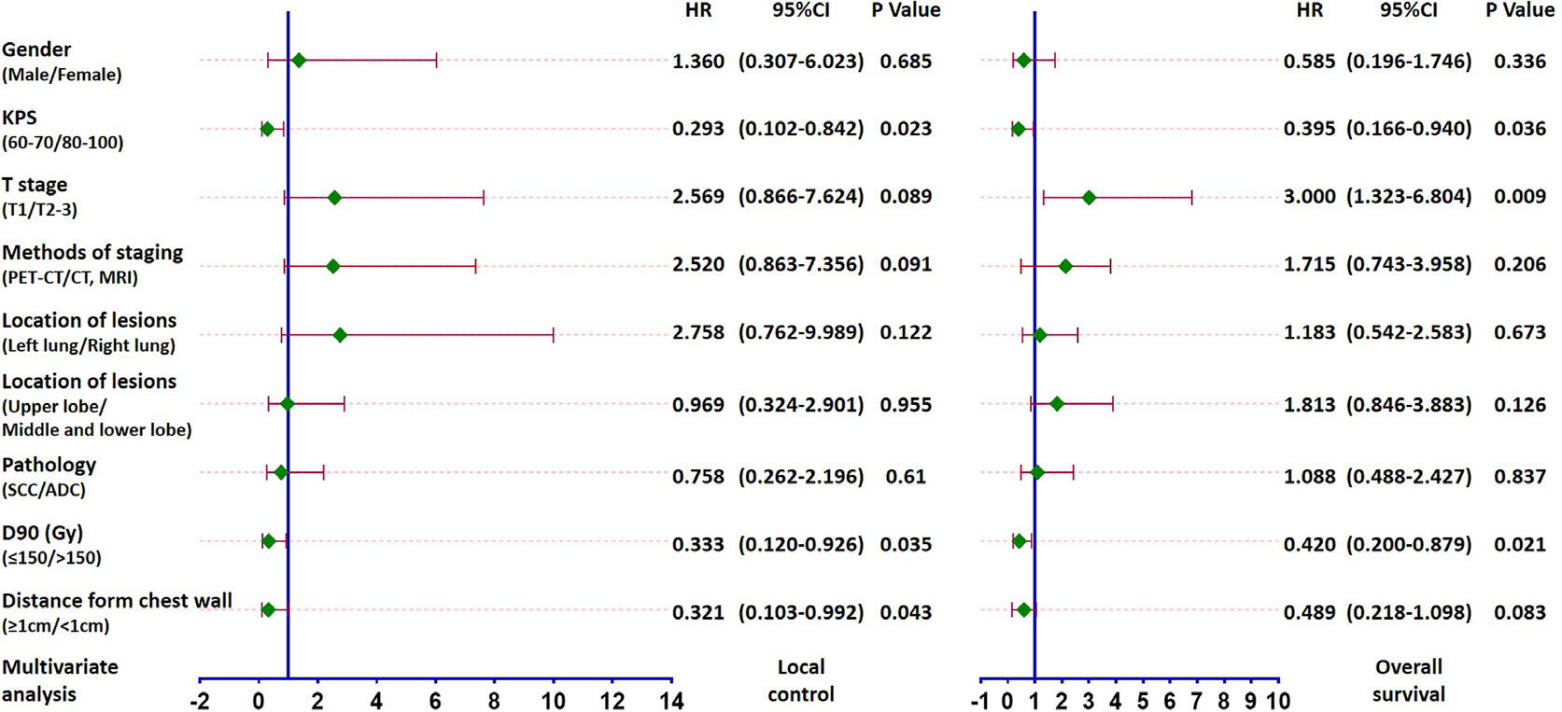
Forest plot based on multivariate analysis of local control and survival: KPS score, GTV D90, and distance between the lesion and chest wall were independent factors for local control and KPS score, T stage and GTV D90 were independent factors for survival (all P < 0.05).

On univariate analysis, the factors significantly associated with better overall survival rate were KPS score 80–100, T1 stage, GTV D90 ≥150 Gy, and the distance between the lesion and chest wall was < 1 cm (all *P* < 0.05) (**Table 2**). The 5-year survival rates were 61.3%, 71.4%, 68.1%, and 66.9%, respectively. The factors significantly associated with better local control rate were KPS score 80–100, T1 stage, the lesion was located in the left lobe, GTV D90 ≥150 Gy, and the distance between the lesion and chest wall was < 1 cm (all *P* < 0.05) (**Table 2**). The 5-year local control rates were 81.3%, 86.6%, 90.2%, 87.4%, and 88.7%, respectively. If the dose was further subdivided, the 5-year local control rates of patients with GTV D90 <150 Gy, 150-180 Gy, and >180 Gy were 58.5%, 78.8%, and 96.3% (*P* = 0.005), respectively (**Figure 2G**). Moreover, the 5-year survival rates of patients with GTV D90 <150 Gy, 150-180 Gy, and >180 Gy were 36.1%, 70.7%, and 66.7% (*P* = 0.023), respectively (**Figure 2H**). The 5-year survival rates of male and female patients were 49% and 72.1%, respectively. The difference was close to statistically significant (*P* = 0.052). On multivariate analysis, KPS score, T stage, and GTV D90 were independent factors for survival, and KPS score, GTV D90, and distance between the lesion and chest wall were independent factors for local control (*P* < 0.05) (**Figures 2**, **3**).

**Table 2:** Univariate analysis of factors associated with local control and survival.

Factors	N	Local control rates			p	Overall survival rates			p
		1-year (%)	3-year (%)	5-year (%)		1-year (%)	3-year (%)	5-year (%)	
Gender					0.188				0.052
Male	72	86.7	74.3	71.9		95.7	62.9	49.0	
Female	27	95.8	86.7	86.7		100.0	90.3	72.1	
KPS					0.006				0.016
60-70	15	73.3	59.3	47.4		93.3	51.9	22.2	
80-90	84	92.3	81.3	81.3		96.1	74.0	61.3	
T stage					0.023				0.001
T1	46	95.2	86.6	86.6		97.6	84.3	71.4	
T2-3	53	84.0	70.1	66.0		96.0	58.0	37.2	
Methods of staging					0.320				0.523
PET-CT	78	89.1	81.2	78.9		95.9	71.5	57.9	
CT/MRI	21	89.2	64.7	64.7		100	64.5	43	
Location of lesions					0.011				0.300
Left lobe	41	97.2	90.2	90.2		94.7	71.4	62.6	
Right lobe	58	83.5	68.9	66.1		98.1	69.0	48.7	
Location of lesions					0.572				0.211
Upper lobe	63	87.8	75.9	73.4		98.3	72.9	59.3	
Middle/lower lobe	36	91.3	80.1	80.1		94.1	65.3	46.0	
Pathology					0.242				0.114
SCC^*^	45	83.3	70.8	70.8		95.2	56.3	52.0	
ADC^*^	54	94.0	82.7	79.7		98.0	80.8	57.3	
D90					0.004				0.009
≤150 Gy	42	84.8	63.8	59.8		97.4	63.3	36.7	
>150 Gy	57	92.2	87.4	87.4		96.3	74.8	68.1	
Distance from CW*					0.012				0.016
≥1cm	60	82.9	69	64.9		96.2	56.8	46.0	
<1cm	39	97.4	88.7	88.7		97.4	86.3	66.9	

^*^SCC, squamous cell carcinoma; ADC, adenocarcinoma; CW, chest wall.

In terms of complications, the number of needles and the distance between the lesion and chest wall was significantly correlated with the incidence of pneumothorax. The incidence of pneumothorax in patients with ≤ 6 needles (n=47) and > 6 needles (n=52) was 36.2% and 57.7%, respectively, and the incidence of pneumothorax in patients with lesions <1 cm (n=39) and ≥1cm (n=60) from the chest wall was 33.3% and 56.7%, respectively. The differences were statistically significant (*P*=0.044 and 0.026, respectively). The postoperative dose (GTV D90) of 2 patients with radiation pneumonitis was 144 Gy and 208.8 Gy, respectively.

## Discussion

The standard treatment for T1–3N0M0 (Ia–IIb) NSCLC is surgery and radical EBRT (including SABR) (1). Currently, few clinicians would choose L-SABT as a treatment for early NSCLC patients, even if surgery is infeasible. However, L-SABT treatment maybe a reasonable choice, with several unique advantages. First, the half-valence layer of I-125 seeds in tissue is 1.7 cm. The dose to the tumor target area is extremely high, while the dose to the surrounding normal tissue is low. The dose rate of γ-ray is low (8–10 cGy/h), which is theoretically less likely to damage normal tissue (15). Second, the distribution of seeds in the lesion can be adjusted during the operation to ensure that the dose distribution in the target area conforms to the actual tumor outline; thus, with L-SABT it is possible to achieve intensity-modulated radiation in a true sense, with better treatment efficacy and less risk of adverse reactions. Third, the γ-rays generated continuously by implanted seeds kill tumor cells over a long period, which could well overcome the errors caused by internal target volume (ITV) and planning target volume (PTV) during treatment. The dose management is more accurate. Fourth, the patient only needs to be hospitalized once, and so the treatment experience is better. Finally, the operation and required facilities (software and hardware) are relatively simple and a linear accelerator is not required, which means the cost of treatment is relatively low and the procedure can even be performed in a primary care hospital. Therefore, in the real-world clinical practice, there are still a few patients with early-stage NSCLC who cannot be operated on who have received L-SABT treatment. This retrospective study focuses on the cases from 8 medical centers over a period of 10 years (2010-2020), which belongs to a relatively large sample study in terms of L-SABT treatment of early NSCLC. We hope that the data of efficacy and toxicity obtained in this study can help clinicians to understand and to evaluate the safety and effectiveness of this treatment for early-stage NSCLC.

In this study, the 3-year and 5-year local control rate was 77.5% and 75.7% (86.6% for T1 patients). In the study of Martinez-Monge et al., they treated 7 T1N0M0 NSCLC patients with L-SABT, a good local control was also found. After median follow-up of 13 months (range, 4.6–41.0+ months) they found no local or regional recurrence (16). Although studies on SABR showed the 3-year local control rate to be ≥90% (17–19), in some studies with long-term follow-up the 5-year local control rate was about 80% (20–22). It could be considered that local control rate of L-SABT may be comparable with SABR. In this study, a large dose span has been observed (100-278.8 Gy), D90 was the independent predictor of local control and overall survival. The local control rate was higher in patients with high D90. For patients with D90 >180 Gy, the local control rate was even as high as 96.3% at 5 years. Moreover, there were no grade 3 or above toxicities. It is suggested that if the dose is further increased, the effect of SABR may be reached or exceeded. In non-prostate tumors, few studies have reported the relationship between the dose of radioactive seeds and local control. In our study, as expected, patients with higher dose had better local control. Although, according to the ABS (American Brachytherapy Society) guidelines, the recommended dose for single application is 100–125 Gy (23), our patients received higher doses on the premise that better efficacy could be achieved without increasing the risk of toxicity. In univariate analysis, T stage and D90 were the influencing factors of local control, but in multivariate analysis, only D90 was the independent influencing factor, suggesting that dose may play a more important role in local control than T stage. In the future, relevant dosimetry studies should be carried out to further clarify the optimal tumor control dose. In addition, in our study, patients with lesions that were close to the chest wall had better local control and survival. Compared with SABR and thermal ablation, for which we need to pay attention to the chest wall toxicity and reaction, L-SABT may be more suitable for this group (lesions <1 from the chest wall). In our sample, the 1-year, 3-year, and 5-year survival rates in this study were 96.7%, 70.1%, and 54.4%, respectively. The 5-year survival rate of T1 patients was 71.4%. Distant metastasis was still the main cause of failure (23.2%), which was similar to that of SABR. Univariate analysis showed that T stage, GTV D90, and distance between the lesion and chest wall were influence factors for survival. However, in multivariate analysis, distance between the lesion and chest wall did not become an independent factor for survival, which may be related to the fact that most of the patients with lesions < 1cm from the chest wall were more likely to be T1 and could reach higher doses. Thermal ablation can also be used as a potentially effective treatment for early NSCLC that is not suitable for surgery (1). The 1-year and 3-year local control rates can reach 86.0%-96.0%, 64.0% - 77.5%, respectively, and the 1-, 3-, and 5-year survival rates can reach 70.0%-96.0%, 43.0%-67.1%, and 16.0%-36.3%, respectively (24–27). This study has similar local control rates and even better survival rates compared with that.

The main consideration limiting the use of external beam radiation dose is radiation-related adverse reaction. About 5%–10% of patients treated with SABR suffer grade 3 or above toxicities and side effects (pneumonia, chest pain, hemoptysis, and so on) (18, 20, 28). In some studies, the proportion is as high as 15%–30% (21, 22, 29). The incidence of radiation toxicities and side effects is very low with L-SABT. In some studies on head and neck tumors and chest wall tumors, the incidence of grade 1 and 2 skin/mucosa adverse effects was 7%–30%; no grade 3 or 4 toxicities were reported, although most of the patients had received radiotherapy previously (6, 30). One previous study has shown that L-SABT could increase the risk of esophageal fistula and tracheal fistula if the lesion is located in the mediastinum (31). However, the mediastinum is not invaded in early lung cancer. The present study also showed low toxicity with L-SABT. Two patients (with D90 of 144 Gy and 208.8 Gy, respectively) had grade 2 radiation pneumonia which improved with medication. However, it should be noted that there is limited data, and the dose/toxicity relationship of L-SABT needs to be further investigated.

Unlike external radiotherapy, L-SABT treatment is invasive and so there may be puncture-related complications. In the present study, the most common complication was pneumothorax, with an incidence of 47.5%. In previous studies about percutaneous lung biopsy, the incidence of pneumothorax was mostly <30%, with <10% of patients needing drainage (32–34). Pneumothorax is also the most common complication in the ablation therapy of early-stage lung cancer, with an incidence of 12% - 63.8% (24–27). The incidence of pneumothorax was higher in our study, probably because of the longer operation time of L-SABT and the higher number of puncture needles used when compared with the previous studies. The incidence of puncture complications was related to many factors such as operation time, needle adjusting frequency, lesion size, and so on (32–35). In the analysis of influencing factors in this study, the incidence of pneumothorax was lower in patients with ≤ 6 needles and < 1cm from the lesion to the chest wall, which again suggested that patients with smaller lesions and lesions that were closer to the chest wall might be more suitable for L-SABT treatment. Most other complications were mild and had low incidence. In general, the safety of L-SABT treatment is considered acceptable according to the data of this study.

The main shortcoming of previous L-SABT was that the treatment depends too much on personal experience and the quality of implantation was difficult to guarantee. Therefore, the dose range in our study was relatively large (100–278.8 Gy). Recently, we developed a 3D printing template (3DPT) technology. It has been proved that there is favorable consistency between the postoperative dose and preoperative dose through 3DPT combined with CT guidance, which can provide quality assurance for the L-SABT study (36). In addition, seeds strand (37), navigation technology (38), and robotics technology (39), which can be applied to L-SABT, are expected to further improve the accuracy and efficiency of treatment and reduce the incidence of the complications, so that L-SABT in the local treatment of cancer may be more standardized and widely used in the future.

The limitations of the study were as follows: (1) In clinical practice there are very few patients with early NSCLC who do not undergo surgery or SABR and receive L-SABT instead, so the sample size was small (difficult to form big data). (2) Because of the retrospective study, the further details of reasons for not performing surgery or EBRT were not recorded. (3) The toxicities information was not collected in detail enough, and the data obtained are only for reference. (4) Because most of the patients and their families who received L-SABT were not willing to treat their diseases actively enough, the patients’ combined treatments (such as chemotherapy, targeted therapy, and so on) was very seldom and difficult to count in detail, which may potentially affect the treatment outcome. It is hoped that there will be an opportunity for future prospective study to further clarify the results of the study.

## Conclusion

The efficacy of L-SABT was closed to SABR and had low radiotoxicity in the treatment of inoperable early-stage NSCLC. Despite some unique advantages, due to the invasive operation and high incidence of pneumothorax, it is not suitable to be recommended as superior to SABR. If EBRT is not available, it may be used as one of the treatment options under the condition of full multidisciplinary evaluation and informed notification. Especially for patients with T1 lesions and < 1cm from the chest wall, L-SABT may become a more suitable potential candidate, and prescription dose greater than 150 Gy (preferably greater than 180 Gy) may bring better results. With the progress of implantation equipment and technology, if we can further improve the quality of operation and reduce the incidence of complications, L-SABT has the potential to become one of the competitive treatment methods.

## Data Availability Statement

The raw data supporting the conclusions of this article will be made available by the authors, without undue reservation.

## Ethics Statement

The studies involving human participants were reviewed and approved by Peking University Third Hospital Medical Science Research Ethics Committee.

## Author Contributions

1. Guarantor of integrity of the entire study: JW, XQH and XKH. 2. Study concepts and design: JW and SC. 3. Literature research: ZJ and BH. 4. Clinical studies: QL, CX, MH, YM, ZW, XZ, YS, YW and HH. 5. Experimental studies/data analysis: ZJ, BH, SL, QL, CX, ZW. 6. Statistical analysis: ZJ and SL. 7. Manuscript preparation: ZJ, BH and SL. 8. Manuscript editing: KZ, RW and SC. All authors contributed to the article and approved the submitted version.

## Conflict of Interest

Authors YM and YS are employed by Staff Hospital of Chengde Iron and Steel Group Co. Ltd.

The remaining authors declare that the research was conducted in the absence of any commercial or financial relationships that could be construed as a potential conflict of interest.

## Publisher’s Note

All claims expressed in this article are solely those of the authors and do not necessarily represent those of their affiliated organizations, or those of the publisher, the editors and the reviewers. Any product that may be evaluated in this article, or claim that may be made by its manufacturer, is not guaranteed or endorsed by the publisher.

## Supplementary Material

The Supplementary Material for this article can be found online at: https://www.frontiersin.org/articles/10.3389/fonc.2021.706242/full#supplementary-material
