# Supplementary material for: Clinical Outcome of CT-Guided Stereotactic Ablative Brachytherapy for Unresectable Early Non-Small Cell Lung Cancer: A Retrospective, Multicenter Study
Source: Front Oncol. 2021 Sep 15;11:706242. doi: 10.3389/fonc.2021.706242 (PMC8480264; doi:10.3389/fonc.2021.706242)
Supplement: Supplementary file 1 [file Table_1.docx]

**Supplementary table (1). Patient and lesion characteristics**

| Characteristics | N | % |
| --- | --- | --- |
| Gender |  |  |
| Male | 72 | 72.7 |
| Female | 27 | 27.3 |
| T stage |  |  |
| T1a (Ia) | 2 | 2.0 |
| T1b (Ia) | 17 | 17.2 |
| T1c (Ia) | 28 | 28.3 |
| T2a (Ib) | 24 | 24.2 |
| T2b (IIa) | 13 | 13.1 |
| T3 (IIb) | 15 | 15.2 |
| Lesion location |  |  |
| Superior lobe of left lung | 23 | 23.2 |
| Inferior lobe of left lung | 18 | 18.2 |
| Superior lobe of right lung | 43 | 43.4 |
| Middle lobe of right lung | 3 | 3.1 |
| Inferior lobe of right lung | 12 | 12.1 |
| Pathological type |  |  |
| Squamous cell carcinoma | 45 | 45.5 |
| Adenocarcinoma | 54 | 54.5 |

**Supplementary table (2). Treatment efficacy**

| Index | N | % |
| --- | --- | --- |
| Failure pattern |  |  |
| Progression-free | 61 | 61.6 |
| LR^*^ | 10 | 10.1 |
| RR^*^ | 4 | 4.1 |
| DM^*^ | 12 | 12.1 |
| LR and RR | 1 | 1.0 |
| LR and DM | 9 | 9.1 |
| RR and DM | 2 | 2.0 |
| Survival |  |  |
| Died | 35 | 35.4 |
| Survived | 64 | 64.6 |

^*^LR: Local Recurrence; RR: Regional Recurrence; DM: Distant Metastasis

**Supplementary table (3). Influence factors of pneumothorax**

| Factors | Pneumothorax | | p |
| --- | --- | --- | --- |
|  | No | Yes |  |
| Distance from CW^*^ |  |  | 0.026 |
| ≥1cm (n=60) | 26 (43.3%) | 34 (56.7%) |  |
| <1cm (n=39) | 26 (66.7%) | 13 (33.3%) |  |
| Number of needles |  |  | 0.044 |
| ≤6 (n=47) | 30 (63.8%) | 17 (36.2%) |  |
| >6 (n=52) | 22 (42.3%) | 30 (57.7%) |  |

^*^CW: Chest wall
